# Evolution and Pathogenicity of the H1 and H3 Subtypes of Swine Influenza Virus in Mice between 2016 and 2019 in China

**DOI:** 10.3390/v12030298

**Published:** 2020-03-09

**Authors:** Yuzhong Zhao, Fachao Sun, Li Li, Ting Chen, Shengliang Cao, Guofei Ding, Fangyuan Cong, Jiaqi Liu, Liting Qin, Sidang Liu, Yihong Xiao

**Affiliations:** 1 Department of Basic Veterinary Medicine, College of Animal Science and Veterinary Medicine, Shandong Agricultural University, 61 Daizong Street, Tai’an 271018, China; ZYZ3578@163.com (Y.Z.); sfc0606@163.com (F.S.); lili06001@163.com (L.L.); sdndcsl@163.com (S.C.); 18706385381@163.com (G.D.); Fangyuan_Cong@163.com (F.C.); WCLiujiaqi0822@163.com (J.L.); Liusid@sdau.edu.cn (S.L.); 2 Shandong Provincial Key Laboratory of Animal Biotechnology and Disease Control and Prevention, Shandong Agricultural University, Tai’an 271018, China; 3 Shandong New Hope Liuhe Group Co., Ltd., Qingdao 266100, China; chen04205149@163.com (T.C.); qinlt2013@163.com (L.Q.)

**Keywords:** swine, influenza virus, subtypes, genotypes, pathogenicity

## Abstract

Pigs are considered a “mixing vessel” that can produce new influenza strains through genetic reassortments, which pose a threat to public health and cause economic losses worldwide. The timely surveillance of the epidemiology of the swine influenza virus is of importance for prophylactic action. In this study, 15 H1N1, one H1N2, and four H3N2 strains were isolated from a total of 4080 nasal swabs which were collected from 20 pig farms in three provinces in China between 2016 and 2019. All the isolates were clustered into four genotypes. A new genotype represented by the H1N2 strain was found, whose fragments came from the triple reassortant H1N2 lineage, classical swine influenza virus (cs-H1N1) lineage, and 2009 H1N1 pandemic virus lineage. A/Sw/HB/HG394/2018(H1N1), which was clustered into the cs-H1N1 lineage, showed a close relationship with the 1918 pandemic virus. Mutations determining the host range specificity were found in the hemagglutinin of all isolates, which indicated that all the isolates had the potential for interspecies transmission. To examine pathogenicity, eight isolates were inoculated into 6-week-old female BALB/c mice. The isolates replicated differently, producing different viral loadings in the mice; A/Swine/HB/HG394/2018(H1N1) replicated the most efficiently. This suggested that the cs-H1N1 reappeared, and more attention should be given to the new pandemic to pigs. These results indicated that new reassortments between the different strains occurred, which may increase potential risks to human health. Continuing surveillance is imperative to monitor swine influenza A virus evolution.

## 1. Introduction

The swine influenza, caused by the swine influenza virus (IAV-S), is an acute respiratory disease in pigs, which is characterized by fever, labored breathing, and coughing [1]. Swine are considered as a “mixing vessel” for human, avian, and swine influenza viruses, in which new IAV can be produced through genetic reassortments because of the presence of a mammalian-like receptor, α2–6 linked sialic acids (SIA), and avian-like receptor α2–3 linked SIA in porcine respiratory epithelial cells [2]. The new reassorted strains can then be transmitted to other species, expanding the lineage infection and endangering animal and human health [3]. 

IAV is a negative-sense, single-stranded RNA virus in the Orthomyxoviridae family [4]. The genome consists of eight RNA strands in the negative-sense orientation, encoding at least 17 proteins (PB2, PB1, PB1-F2, PB1-N40, PA, PA-N155, PA-X, PA-N182, HA, NP, NA, MP1, MP2, M42, NS1 NS2, and NS3) [5]. IAVs are classified into subtypes based on the antigenic differences of the two major surface glycoproteins, hemagglutinin (HA) and neuraminidase (NA). At present, three predominant viral subtypes have been detected, including H1N1, H1N2, and H3N2 [6,7]. In human history, a deadly virus known as the Spanish flu infected approximately 500 million people in 1918 [8], and the classical swine influenza virus (cs-H1N1) likely entered the swine population simultaneously in North America [9]. In Europe, the cs-H1N1 subtype was predominant in pigs until it was replaced by the avian-like H1N1 subtype IAV-S, in 1979 [10]. In China, the cs-H1N1 viruses were recognized as enzootic in 1974. The swine human-like H3N2 viruses (hl-H3N2), which were first reported in Taiwanese pigs in 1969, are antigenically similar to some human virus strains [11]. Due to the rapid evolution of IAV-S, in 1998 in North America, a triple reassortant gene (TRIG) appeared in the new H3N2 viruses (tr-H3N2) containing gene segments from human, avian, and swine viruses [12]. The IAV-S reassortant strain that originated from the cs-H1N1 and hl-H3N2 subtypes were found to pose a direct threat to the human population [13,14,15].

The H1N2 subtype, IAV-S, was first isolated in Japanese pigs in 1978 [16] and reassorted with cs-H1N1 and human H3N2 viruses, and rapidly spread to other areas [17,18,19,20,21]. Among the isolates, at least six different H1N2 genotypes were found, including two reassortants between the tr-H3N2, and cs-H1N1 virus; three reassortants between tr-H1N2, the Eurasian avian-like H1N1 (ea-H1N1) swine virus, and the H9N2 swine virus; and one reassortant between H1N1, the hl-H3N2, and the cs-H1N1 virus [22]. In 2009, there was a pandemic of a new influenza strain, the 2009 H1N1 pandemic virus (H1N1pdm09), which originated in Mexico and was produced by quadruple genetic reassortment [23]. Reassortant viruses containing the internal genes of the H1N1pdm09 have been relatively stable in swine [24].

Although commercial IAV-S vaccines are available, their effectiveness is heavily dependent on the antigenic match of the vaccine strains to the circulating virus strains. IAV-S is also endowed with the perfect ability to undergo antigenic drift and antigenic shift to avoid host immunity [25]. Thus, it is crucial to obtain timely information of the circulating strains. In China, due to low biosecurity, the prevalence of IAV-S coexistence of various subtypes and different genotypes of the same subtype complicates matters further. Thus, it is important to survey the genomic and molecular changes of IAV-S to identify potential vaccine candidates. 

To obtain timely information on the epidemiology of IAV-S, a total of 4080 nasal swabs were collected from pigs in China from 2016 to 2019. The complete viral genomes were sequenced, phylogenetically analyzed, and the pathogenicity of the viruses was evaluated in BALB/c mice. These results will deepen our understanding of the epidemiology of IAV-S in China and provide information for the control of IAV-S infection and human public health.

## 2. Materials and Methods

### 2.1. Sample Collection

A total of 4080 nasal swabs were sampled from 20 pig farms in three provinces in China between 2016 and 2018 (Table 1). One swab was collected for each pig. A few pigs showed signs of respiratory illness, including fever, nasal discharge, sneezing, and low appetite. Pig farms without clinical signs were also sampled. The nasal swabs were stored at −70 °C in phosphate-buffered saline (PBS) with penicillin (2000 U mL^−1^) and streptomycin (2000 U mL^−1^). 

### 2.2. Virus Isolation and DNA Sequencing

The nasal swabs were thawed and centrifuged at 5000× *g* for 3 min at 4 °C. The supernatant was then collected (0.2 mL) and inoculated into two 10-day-old SPF chicken embryos through the allantoic cavity. The inoculated chicken embryos were incubated at 37 °C for 72 h. After three blind passages, embryo allantoic fluids were collected and used for the hemagglutination (HA) assay. The samples with chicken blood agglutination activity were saved and the total RNA was extracted according to the instructions of the Viral RNA Mini Kit (Tiangen, China). The RNA was reverse-transcribed into cDNA by Uni12 universal primers (5′-AGCAAAAGCAGG-3′) using the ReverTra Ace qPCR RT Kit (TOYOBO, Japan). Complete viral genomes were amplified according to Hoffmann’s method [26]. The PCR products were purified by 0.8% agarose gel electrophoresis and sequenced at Shanghai Sangon Biotech Co., Ltd. (Shanghai, China).

### 2.3. Virus Purification and Titration

The isolated strains were purified using 10-day-old SPF chicken embryos by inoculation with 0.1 mL of supernatant diluted with 10^2^–10^4^-fold. The viral titrations were measured by an egg-infectious dose 50% (EID50) using three 10-day-old SPF chicken embryos with 0.1 mL of each supernatant, diluted by 10^1^−10^9^-fold. After incubating at 37 °C for 48 h, the EID_50_ titer in the allantoic fluid was determined according to the method of Reed—Muench. For viral titration in organs, tissues were homogenized by 1:10 dilution with sterile PBS (containing penicillin and streptomycin, 2000 U/mL) and centrifuged at 8000× *g* for 5 min at 4 °C. The supernatant was collected and inoculated into chicken embryos as described above.

### 2.4. Phylogenetic Analysis

The fragments of the IAV-S were aligned and analyzed using the MegAlign program in the DNAStar package (DNASTAR, Madison, USA). Complete genomes of 256 A/H1 and 67 A/H3 swine influenza viruses isolated in China were downloaded from the Influenza Virus Resource at the National Center for Biotechnology Information (NCBI) (http://www.ncbi.nlm.nih.gov/genomes/FLU/FLU.html) and the GISAID database on 10 February 2019. Repetitive sequences in the two databases were removed by matching strain names in Bioedit v7.1.3.0 [27]. The remaining sequences were combined with reference sequences, as described previously [28,29,30], and those generated in the present study. The eight datasets corresponding to the eight gene segments of the influenza A virus were first aligned using MAFFT v7.450 [31] and then adjusted manually in Bioedit v7.1.3.0 [27]. Phylogenetic analysis was conducted using RAxML v8.2.9 with the GTRGAMMA applied as a nucleotide substitution model [32] and 1000 bootstrap replicates. Trees were visualized using FigTree (http://tree.bio.ed.ac.uk/software/figtree/).

### 2.5. Replication and Pathogenicity of IAV-S in Mice

To evaluate the replication and pathogenicity, eight isolates (A/Swine/SD/LY236/2016(H1N1), A/Swine/HN/NY127/2017(H1N1), A/Swine/HB/HG394/2018(H1N1), A/Swine/HN/NY125/2016(H3N2), A/Swine/HN/NY426/2016(H3N2), A/Swine/HN/NY428/2016(H3N2), A/Swine/HN/NY430/2016(H3N2), and A/Swine/SD/LY396/2016(H1N2) represented by different genotypes were selected to learn the pathogenicity in mice. Fifty-four 6-week-old female BALB/c mice were randomly divided into nine groups (including eight virus-infected groups and one mock-infected group). The mice were inoculated intranasally with 10^6^ EID_50_ of purified IAV-S after anesthetizing with CO_2_, which was approved by the Animal Care and Use Committee of Shandong Agricultural University. The project identification code is 2017-041, which was approved on 12 April 2017 (12/04/2017). Three mice in each group were sacrificed at 3 days post-infection (DPI), and tissues, including the brain, nasal turbinate, lung, spleen, and kidney, were collected and maintained at −70 °C for virus titration, or fixed in 4% neutral formaldehyde for pathological observations. The remaining mice were monitored for 14 days to assess body weight loss and mortality. A weight-loss rate of more than 30% was reported as a death. All surviving mice were euthanized by CO_2_ at 14 DPI.

## 3. Results

### 3.1. Virus Isolation and Phylogenetic Analysis

**Figure 1:**
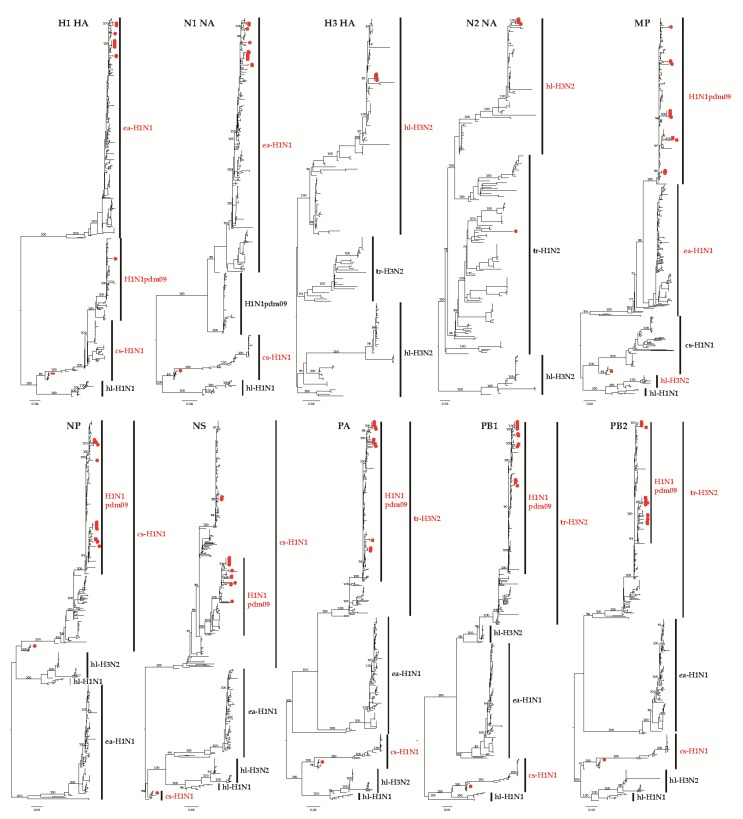
Phylogenetic trees constructed using 20 isolates and the H1 HA (*n* = 515), H3 HA (*n* = 184), N1 NA (*n* = 414), N2 NA (*n* = 240), MP (*n* = 460), NS (*n* = 454), PA (*n* = 432), PB1 (*n* = 440), and PB2 (*n* = 437) gene segments available from GenBank. The 20 new swine influenza virus (IAV-S) isolates are marked with red dots.

The supernatant of nasal swab extracts were inoculated into 10-day-old, specific-pathogen-free (SPF) chicken embryos and passaged blindly three times. Twenty strains, including 15 H1N1, one H1N2, and four H3N2 IAV-S subtypes were successfully isolated (Table 1). The results showed that different subtypes of IAV-S were circulating in China and H1N1 was the dominant subtype. The complete genomes of all isolates were sequenced, and then phylogenetic analysis was performed. Compared with 256 reference H1 viruses isolated during 2002–2018, distinct clusters were formed within the phylogenetic trees corresponded to ea-H1N1, H1N1pdm09, cs-H1N1, and hl-H1N1 (Figure 1). In this study, Of the 16 H1 influenza viruses isolated from 2016 to 2018, 14 were in the ea-H1N1 lineage, one (A/Swine/HB/HG394/2018(H1N1)) in the cs-H1N1 lineage, and one (A/Swine/SD/LY396/2016(H1N2)) in the H1N1pdm09 lineage. The nucleotide similarity between the 16 H1 fragments was 72.9–100%. Compared with 67 H3 viruses isolated during 2002–2019, the HA fragment of our four H3N2 viruses were in the lineage of hl-H3N2 and shared 99.8–100% identity at the nucleotide level. Similarly to the HA fragments, 14 N1 NA fragments were clustered in the ea-H1N1 lineage and one (A/Swine/HB/HG394/2018(H1N1)) N1 NA fragment was clustered in the cs-H1N1 lineage (Figure 1) with nucleotide similarities of 81.8–100% among the 15 strains. N2 fragments of the four H3N2 strains were from the hl-H3N2 lineage, while the N2 fragment of the H1N2 strain was clustered into the tr-H1N2 lineage. The nucleotide similarity of the four N2 fragments from H3N2 was 99.6–100% and shared 72.9–100% identity at the nucleotide level between all five N2 fragments. The results also showed that the PB2, PB1, PA, MP, and NP fragments of the 20 strains showed the same clustering pattern and were clustered into the H1N1pdm09 and cs-H1N1 lineages. The PB2, PB1, PA, MP, and NP fragments of all isolates were clustered into the H1N1pdm09 lineage, except those from A/Swine/HB/HG394/2018(H1N1), which were clustered into the cs-H1N1 lineage. The nucleotide similarities of PB2, PB1, PA, MP, and NP of 20 isolates were 96–100%, 96.2–100%, 96.2–100%, 95.9–100%, and 95.8–100%, respectively. The fragments of PB2, PB1, PA, MP, and NP of A/Swine/HB/HG394/2018(H1N1) were all clustered into the lineage of cs-H1N1. All the PB2, PB1, PA, MP, and NP fragments of all 20 viruses shared 85.1–100%, 81.4–100%, 83.3–100%, 88.8–100%, and 87.4–100% of nucleotide similarities. The NS fragments of 16 H1 subtype viruses were clustered into the H1N1pdm09 lineage, and that of four H3N2 subtype viruses were clustered into the cs-H1N1 lineage. The nucleotide homology among the 20 NS fragments was 86.3–100%. 

### 3.2. Genotyping Analysis

According to the phylogenetic analysis of the gene segments, the 20 isolates were divided into four genotypes, 1, 2, 3, and 4 (Table 2). Fourteen out of 15 H1N1 strains carried TRIG, with fragments from the ea-H1N1 lineage (HA and NA fragments), the cs-H1N1 lineage (NS fragment), and the H1N1pdm09 lineage (PB2, PB1, PA, NP, and M fragments). All fragments of A/Swine/HB/HG394/2018(H1N1) belonged to the cs-H1N1 lineage. The H3N2 strains were double-reassortant, with fragments from the hl-H3N2 lineage (HA and NA fragments), and H1N1pdm09 lineage (PB2, PB1, PA, NP, M, and NS fragments). The H1N2 strain was a TRIG strain, with fragments from the tr-H1N2 lineage (NA fragment), cs-H1N1 lineage (NS fragment), and H1N1pdm09 lineage (PB2, PB1, PA, HA, NP, and M fragments). It is worth noting that the 19 strains contained five H1N1pdm09-originating internal genes (PB2, PB1, PA, NP, and M fragments), indicating the high prevalence of the H1N1pdm09 subtype. The H1 subtype also had an NS fragment derived from cs-H1N1. In the H3 subtype, six internal genes were from H1N1pdm09.

### 3.3. Molecular Characteristics Analysis of the Isolates

To understand the molecular characteristics of the isolated IAV-S strains, key amino acid sites were analyzed. HA was initially translated into a single molecule, HA0, which was then cleaved by host proteases into HA1 and HA2, where HA1 and HA2 are covalently linked by a disulfide bond [33]. The cleavage sites of all isolated H1 subtype IAV-S were PSIQSR/G, while those of the H3 subtype IAV-S were PEKQTR/G. Such cleavage sites are characteristic of low pathogenicity of avian IAV (Table 3). The receptor-binding site of the HA protein determines the receptor-binding characteristics of IAV-S and is directly related to host tropism [34]. The receptor binding sites of the HA protein from our isolated H1 subtype IAV-S were at the V155, N159, H183, D/N/V190, D/E/G225, Q/R226, A/E227, and G228 positions, respectively (Table 3). All of the H1 subtype IAV-S that we isolated exhibited HA T155V, T159N, and E190D mutations, except for A/Swine/SD/LY236/2016(H1N1), A/Swine/HB/HG332/2018(H1N1), and A/Swine/HB/HG354/2018(H1N1). Those results indicated that most of the HA genes from the isolated H1N1 strains may have the potential for transmission between avian and pig species. All H1 subtype IAV-S contained 225E and 190D mutations, except for A/Swine/SD/LY236/2016(H1N1), A/Swine/SD/LY396/2016(H1N2), A/Swine/HN/NY127/2017(H1N1), A/Swine/HB/HG332/2018(H1N1), A/Swine/HB/HG354/2018(H1N1), and A/Swine/HB/HG394/2018(H1N1), indicating that those strains could potentially infect human beings. The receptor-binding sites of the isolated H3 subtype IAV-S were at amino acid sites T155, Y159, H183, N190, D225, I226, S227, and S228 (Table 3). Our hl-H3N2 virus has characteristic residues found in human-adapted seasonal H3N2 viruses, including 226I and 228S [35]. H1 HAs had potential N-glycosylation sites, including 14NST, 26NVT, 57NCS, 90NGT, 277NCT, 290NTS, 484NGT, and 543NGS. Only A/Swine/SD/LY236/2016(H1N1) had glycosylation sites at 57NCS, and A/Swine/SD/LY396/2016 had glycosylation sites at 90NGT and 290NTS. Four H3 HAs contained six potential N-glycosylation sites, including 22NGT, 63NCT, 126NWT, 246NST, 285NGS, and 483NGT. Viruses with lysine at position 627 were pathogenic in mice, whereas those with glutamic acid were nonpathogenic in these animals [36]. Only A/Swine/HB/HG394/2018(H1N1) possessed a K at position 627, an A at position 271, and an R at position 591 of PB2. Twenty strains of the influenza virus contained D at position 701 with no mutations (Table 2). H274Y in the N1 gene, and E119V and R292K in the N2 gene served as reference sites for neuraminidase inhibitor (NAI) resistance [37,38]. No isolates had NAI-resistant amino acids (Table 3), indicating that these viruses are sensitive to NAIs, such as oseltamivir. In the M2 genes which contained the key amino acid target of amantadine drugs [39], the key amino acid sites, L26, V/I27, A30, N/S31, and G34 were found in our isolated H1 subtype, while L26, I27, A30, N31, and G34 were found in the H3 subtype (Table 3). 

### 3.4. Pathogenicity of the Isolates in Mice

**Figure 2:**
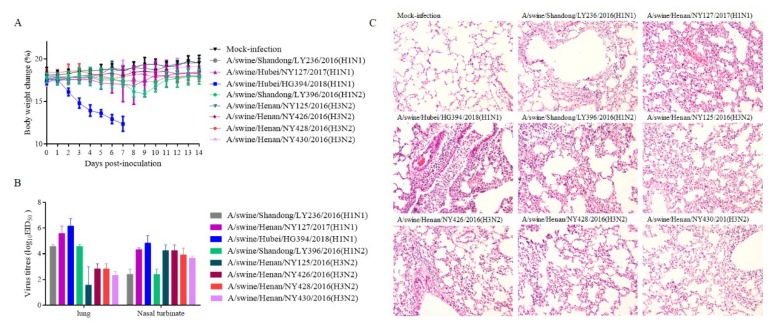
Pathogenicity of the isolated IAV-S in mice. (**A**) Mouse body weights were monitored daily for 14 days. The values represent the average scores of overall body weight loss, compared with the initial body weight ± standard deviation (SD). (**B**) Viral titers in the lungs and nasal turbinate of the infected mice (*n* = 3) at 3 DPI. (**C**) Histopathology analysis of lungs. Lungs of the infected mice were fixed with formalin, embedded in paraffin, and stained with hematoxylin and eosin and observed under a microscope with 200× magnification.

To evaluate the replication and pathogenicity, eight isolates, including three H1N1 (A/Swine/SD/LY236/2016, A/Swine/HN/NY127/2017, and A/Swine/HB/HG394/2018), four H3N2 subtypes (A/Swine/HN/NY125/2016, A/Swine/HN/NY426/2016, A/Swine/HN/NY428/2016, A/Swine/HN/NY430/2016), and the H1N2 subtype A/Swine/SD/LY396/2016, represented by different genotypes, were inoculated into 6-week-old female BALB/c mice with 10^6^ EID_50_. Compared to the control group, mice infected with A/Swine/HN/NY428/2016(H3N2), A/Swine/SD/LY236/2016(H1N1), A/Swine/HN/NY430/2016(H3N2), A/Swine/HN/NY125/2016(H3N2), A/Swine/HN/NY127/2017(H1N1), or A/Swine/HN/NY426/2016(H3N2) exhibited no clinical signs. However, the mice inoculated with A/Swine/HB/HG394/2018 exhibited ruffled fur, severe inappetence, emaciation, and lost up to 29.45% body weight by day 7 DPI, resulting in 100% death (Figure 2A). The mice infected with A/Swine/SD/LY396/2016(H1N2) lost up to 10.42% body weight by 9 DPI, which then increased slowly until it was restored at 11 DPI (Figure 2A). At 3 DPI, three mice in each group were sacrificed and tissues were collected for detection of viral loads. The results showed that different viral loads in the same organs were caused by different strains. Viral loads in the lung and nasal turbinate of mice infected with A/Swine/HB/HG394/2018 were higher (Figure 2B) than that of other H1N1 subtypes. In mice infected with the four H3N2 subtype viruses of A/Swine/HN/NY428/2016, A/Swine/HN/NY426/2016, A/Swine/HN/NY430/2016, and A/Swine/HN/NY125/2016, a higher titer was found in the nasal turbinate than in the lungs, while in mice infected with A/Swine/SD/LY236/2016(H1N1), A/Swine/SD/LY396/2016(H1N2), A/Swine/HN/NY127/2017(H1N1), and A/Swine/HB/HG394/2018(H1N1) a higher virus load was detected in the lungs than in the nasal turbinate (Figure 2B). No virus was detected in the kidney, brain, and spleen. The histopathological changes in the lungs showed that similar changes of alveolar wall thickening, inflammatory cell infiltration, capillary congestion, and epithelial cell shedding were observed in all infected groups (Figure 2C). The lungs of A/Swine/HB/HG394/2018(H1N1)-infected mice showed more severe changes, with larger amounts of epithelial cell shedding and a larger volume of infiltrating inflammatory cells.

## 4. Discussion

IAV-S, as a zoonotic disease, has an important impact on public health [39]. Since 2009, the reassortant viruses derived from H1N1pdm09 and other swine influenza viruses have been isolated in pigs in some countries [40,41]. Sporadic cases of human infection with such swine-origin novel reassortant viruses have been reported [42]. All these indicated that IAV-S poses a serious threat to the health of human beings. In this study, 20 IAV-S strains, including 15 H1N1, one H1N2, and four H3N2 subtypes were identified, which indicated that different subtypes have been co-circulating in pigs in China (Table 1). Based on the genetic and phylogenetic analyses, four genotypes were clustered (Table 2). Five or six gene fragments of most strains were derived from swine H1N1pdm09, indicating that recently, swine H1N1pdm09 has been the dominant lineage. This provides direct evidence that the H1N1pdm09 internal gene complex has been successfully incorporated into different subtypes of IAV-S. Notably, except for A/swine/Hubei/HG394/2018(H1N1), the fragments of all H1N1 viruses were in the same reassortant pattern—HA and NA from the ea-H1N1 lineage, PB1, PB1, PA, NP, and MP from the H1N1pdm09 lineage, and NS from the cs-H1N1 lineage, which indicated that this may be to the dominant genotypes during their adaption in swine.

Since the isolation of the first IAV-S by Shope from pigs in 1930, these cs-H1N1 viruses likely derived from the 1918 pandemic IAV [43]. Based on the phylogenetic analysis results, A/swine/Hubei/HG394/2018(H1N1) was clustered into the cs-H1N1 lineage, which is closely related with the 1918 pandemic virus (Figure 1). Interestingly, a sub-lineage within the cs-H1N1 lineage was formed. In this sub-lineage, only A/swine/Guangdong/L3/2009(H1N1) are from the mainland of China [44]. Other strains are from the USA or UK, most of which are related to early human influenza. This indicates that the previous IAV-S appeared again, and more attention should be paid to this virus and also on whether a new pandemic to pigs would be caused by the existence of this subtype virus. Eight isolates represented by four different genotypes were inoculated into mice to compare their pathogenicity. The results showed that mice infected with A/Swine/HB/HG394/2018 (H1N1 subtype) experienced rapid weight loss and resulted in 100% death (Figure 2A). Additionally, high viral loads (Figure 2B) were detected in the lung and nasal turbinate from mice infected with A/Swine/HB/HG394/2018(H1N1). Severe interstitial pneumonia was also observed in the lungs of mice with A/Swine/HB/HG394/2018(H1N1) (Figure 2C). Mice infected with other H1 subtypes had high virus titers in their lungs, although such levels caused no clinical signs or obvious weight loss. For H1 subtype strains, the substitutions of E190D are critical for the shift from α-2,3- to α-2,6-linked SIA receptor recognition [45]. In this study, an N/V190 was found in A/Swine/SD/LY236/2016(H1N1), A/Swine/HB/HG332/2018(H1N1), and A/Swine/HB/HG354/2018(H1N1) isolates, while 190D was found in all other H1 fragments (Table 3). 

The presence of specific amino acids in PB2 are important for the replication and virulence of influenza viruses in mammals [46]. E627K or D701N mutations in PB2 are markers for enhanced polymerase activity and viral replication in mammalian cells, as well as the pathogenicity of H7N9 viruses in the BALB/c mouse model [47]. The presence of PB2-627K enhances viral replication and pathogenicity in mammalian model systems, and is associated with lethality in humans infected with H5N1 and H7N7 avian viruses [48]. For the H1N1pdm09 virus, it was reported that the amino acid alanine (A) at position 271 of PB2 enhances the transmissibility of influenza A in ferrets, and arginine (R) at position 591 is important for H1N1pdm09 in mammalian adaptation [49]. Nineteen out of 20 strains of the influenza virus contained E and D at positions 627 and 701, respectively, with no mutations. A lysine (K) at amino acid position 627 was found in A/Swine/HB/HG394/2018 (Table 3). It was reported that R at position 591 of PB2 can compensate for the lack of K at position 627 and confers efficient viral replication to H1N1pdm09 viruses in mammals [50]. In this study, only A/swine/Hubei/HG394/2018(H1N1) possessed K627, T271, and Q591 of PB2, which is characteristic of mammalian influenza viruses and which may be a reason for the high rate of death in mice infected with the A/swine/Hubei/HG394/2018(H1N1) virus.

A glutamic acid (E) at amino acid position 225 was found in all H1 genes, except for A/Swine/SD/LY396/2016(H1N2), A/Swine/HN/NY127/2017(H1N1), and A/Swine/HB/HG394/2018(H1N1). The substitution of glycine (G) for glutamic acid (E) at position 225 (E225G) plays a critical role in the transmission of the ea-H1N1 virus by increasing the efficiency of viral assembly and budding [51]. Thus, the difference in amino acid mutations of the HA gene may explain why H1 subtypes replicated more efficiently in the lung than in the nasal turbinate. For H3 viruses, the substitutions of Q226L and G228S in HA was accepted as a complete switch from α-2,3- to α-2,6-linked receptor-binding specificity [52,53]. In all four H3N2 subtype viruses, only G228S was observed, which indicated that the receptor-binding specificity was partially changed. As a result, all four H3N2 subtype strains replicated less efficiently in the lungs than the nasal turbinate.

The amino acid residue at position 31 of the M2 protein of the 19 out of 20 influenza viruses were all mutated to N, indicating that all such viruses are resistant to amantadine (Table 3). Of the 20 influenza viruses, no sites in the NA protein were linked to NAI resistance, indicating that these viruses are sensitive to NAIs, such as oseltamivir.

## 5. Conclusions

Based on this study, different IAV-S subtypes have been co-circulating in pigs in China. Swine H1N1pdm09 has recently been the dominant lineage. A new genotype of the H1N2 subtype was found. Molecular characteristics analysis indicated that the isolates have the potential for interspecies transmission. A cs-H1N1 strain (A/swine/Hubei/HG394/2018(H1N1)) was isolated, which is closely related to the 1918 pandemic virus. All the isolates replicated differently in mice, and A/swine/Hubei/HG394/2018(H1N1) replicated more efficiently than other subtypes. These results indicate that new reassortments between the different strains occurred, and that continued surveillance is required to monitor the evolution of the swine influenza A virus.

## Figures and Tables

**Table 1:** Information of samples collected for IAV-S isolates.

Province	Farms	Sample Numbers	Collection Time	Strains Abbreviation	Subtypes	Clinical Signs
Henan	1#	300	2016.9	A/Swine/HN/NY426/2016	H3N2	NO
	2#	230	2016.10	A/Swine/HN/NY428/2016	H3N2	NO
	3#	120	2016.10	A/Swine/HN/NY430/2016	H3N2	NO
	4#	140	2016.12	A/Swine/HN/NY125/2016	H3N2	NO
	5#	60	2017.3	A/Swine/HN/NY127/2017	H1N1	Coughing and sneezing
	6#	300	2018.2	A/Swine/HN/NY364/2018	H1N1	NO
Shandong	7#	300	2016.12	A/Swine/SD/LY396/2016	H1N2	NO
	8#	50	2016.12	A/Swine/SD/LY236/2016	H1N1	Fever, coughing, sneezing,
	9#	160	2017.2	A/Swine/SD/LW4/2017	H1N1	Fever, coughing, sneezing
	10#	300	2017.3	A/Swine/SD/LW7/2017	H1N1	NO
	11#	200	2017.3	A/Swine/SD/LW13/2017	H1N1	NO
	12#	200	2017.10	A/Swine/SD/LW21/2017	H1N1	NO
	13#	170	2017.11	A/Swine/SD/LW34/2017	H1N1	NO
	14#	230	2017.11	A/Swine/SD/LW36/2017	H1N1	NO
	15#	200	2017.12	A/Swine/SD/LW37/2017	H1N1	NO
	16#	140	2017.12	A/Swine/SD/LW40/2017	H1N1	Fever, coughing, sneezing
	17#	400	2017.12	A/Swine/SD/TA27/2017	H1N1	Coughing and sneezing
Hubei	18#	280	2018.11	A/Swine/HB/HG332/2018	H1N1	NO
	19#	180	2018.11	A/Swine/HB/HG354/2018	H1N1	NO
	20#	120	2018.12	A/Swine/HB/HG394/2018	H1N1	Fever, coughing, sneezing

NO: No obvious clinical signs. 1 swab = 1 pig.

**Table 2:** The genotypes of all isolates.

Isolate Name	Genotype	Lineage Assigned to Gene Segments							
		PB2	PB1	PA	HA	NP	NA	MP	NS
A/Swine/SD/LY236/2016(H1N1)	1								
A/Swine/SD/LW4/2017(H1N1)									
A/Swine/SD/LW7/2017(H1N1)									
A/Swine/SD/LW13/2017(H1N1)									
A/Swine/SD/LW21/2017(H1N1)									
A/Swine/SD/LW34/2017(H1N1)									
A/Swine/SD/LW36/2017(H1N1)									
A/Swine/SD/LW37/2017(H1N1)									
A/Swine/SD/LW40/2017(H1N1)									
A/Swine/SD/TA27/2017(H1N1)									
A/Swine/HN/NY127/2017(H1N1)									
A/Swine/HB/HG332/2018(H1N1)									
A/Swine/HB/HG354/2018(H1N1)									
A/Swine/HN/NY364/2018(H1N1)									
A/Swine/HB/HG394/2018(H1N1)	2								
A/Swine/HN/NY426/2016(H3N2)	3								
A/Swine/HN/NY428/2016(H3N2)									
A/Swine/HN/NY430/2016(H3N2)									
A/Swine/HN/NY125/2016(H3N2)									
A/Swine/SD/LY396/2016(H1N2)	4								

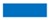
 H1N1pdm09 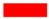 ea-H1N1 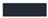 tr-H1N2 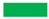 cs-H1N1 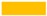 hl-H3N2.

**Table 3:** Receptor binding sites and mutant sites analysis of the isolates.

Virus	Subtypes	HA ^a^			HA^a^								PB2		NA ^b^			M2				
			155	159	183	190	225	226	227	228	271	591	627	701	119	292	274	26	27	30	31	34
A/Swine/SD/LY236/2016	H1N1	PSIQSR/GL	V	N	H	N	E	Q	A	G	A	R	E	D			H	L	I	A	N	G
A/Swine/SD/LW4/2017	H1N1	PSIQSR/GL	V	N	H	D	E	Q	A	G	A	R	E	D			H	L	V	A	N	G
A/Swine/SD/LW7/2017	H1N1	PSIQSR/GL	V	N	H	D	E	Q	A	G	A	R	E	D			H	L	V	A	N	G
A/Swine/SD/LW13/2017	H1N1	PSIQSR/GL	V	N	H	D	E	Q	A	G	A	R	E	D			H	L	V	A	N	G
A/Swine/SD/LW21/2017	H1N1	PSIQSR/GL	V	N	H	D	E	Q	A	G	A	R	E	D			H	L	V	A	N	G
A/Swine/SD/LW34/2017	H1N1	PSIQSR/GL	V	N	H	D	E	Q	A	G	A	R	E	D			H	L	V	A	N	G
A/Swine/SD/LW36/2017	H1N1	PSIQSR/GL	V	N	H	D	E	Q	A	G	A	R	E	D			H	L	V	A	N	G
A/Swine/SD/LW37/2017	H1N1	PSIQSR/GL	V	N	H	D	E	Q	A	G	A	R	E	D			H	L	V	A	N	G
A/Swine/SD/LW40/2017	H1N1	PSIQSR/GL	V	N	H	D	E	Q	A	G	A	R	E	D			H	L	V	A	N	G
A/Swine/SD/TA27/2017	H1N1	PSIQSR/GL	V	N	H	D	E	Q	A	G	A	R	E	D			H	L	I	A	N	G
A/Swine/HN/NY127/2017	H1N1	PSIQSR/GL	V	N	H	D	G	Q	A	G	A	R	E	D			H	L	V	A	N	G
A/Swine/HB/HG332/2018	H1N1	PSIQSR/GL	V	N	H	V	E	Q	A	G	A	R	E	D			H	L	V	A	N	G
A/Swine/HB/HG354/2018	H1N1	PSIQSR/GL	V	N	H	V	E	Q	A	G	A	R	E	D			H	L	V	A	N	G
A/Swine/HB/HG394/2018	H1N1	PSIQSR/GL	V	N	H	D	G	Q	A	G	T	Q	K	D			H	L	V	A	S	G
A/Swine/HN/NY364/2018	H1N1	PSIQSR/GL	V	N	H	D	E	Q	A	G	A	R	E	D			H	L	V	A	N	G
A/Swine/SD/LY396/2016	H1N2	PSIQSR/GL	V	N	H	D	D	R	E	G	A	R	E	D	E	R		L	V	A	N	G
A/Swine/HN/NY426/2016	H3N2	PEKQTR/GI	T	Y	H	N	D	I	S	S	A	R	E	D	E	R		L	I	A	N	G
A/Swine/HN/NY428/2016	H3N2	PEKQTR/GI	T	Y	H	N	D	I	S	S	A	R	E	D	E	R		L	I	A	N	G
A/Swine/HN/NY430/2016	H3N2	PEKQTR/GI	T	Y	H	N	D	I	S	S	A	R	E	D	E	R		L	I	A	N	G
A/Swine/HN/NY125/2016	H3N2	PEKQTR/GI	T	Y	H	N	D	I	S	S	A	R	E	D	E	R		L	I	A	N	G

^a^: Amino acid numbering refers to the HA gene of H3 subtype (GenBank accession #CY008156.1); ^b^: Amino acid numbering refers to the NA gene of N2 subtype (GenBank accession #CY008158.1).
